# Molecular Amplification and Cell Culturing Efficiency for Enteroviruses’ Detection in Cerebrospinal Fluids of Algerian Patients Suffering from Meningitis

**DOI:** 10.3390/v16020170

**Published:** 2024-01-23

**Authors:** Abdelwahab Rai, Zohra Ammi, Dahbia Leila Anes-Boulahbal, Aymen Amin Assadi, Abdeltif Amrane, Oussama Baaloudj, Lotfi Mouni

**Affiliations:** 1 Laboratoire de Gestion et Valorisation des Ressources Naturelles et Assurance Qualité, Faculté SNVST, Université de Bouira, Bouira 10000, Algeria; l.mouni@univ-bouira.dz; 2 Faculté SNVST, Université de Bouira, Bouira 10000, Algeria; microbiologiste95@gmail.com; 3 Laboratoire des Entérovirus, Département de Virologie, Institut Pasteur d’Alger, Annexe de Sidi-Fredj, Alger 16000, Algeria; anes_boulahbal_leila@yahoo.fr; 4 College of Engineering, Imam Mohammad Ibn Saud Islamic University, IMSIU, Riyadh 11432, Saudi Arabia; aaassadi@imamu.edu.sa; 5 Ecole Nationale Supérieure de Chimie de Rennes, University Rennes, CNRS, ISCR-UMR 6226, 35000 Rennes, France; abdeltif.amrane@univ-rennes.fr; 6 Laboratory of Reaction Engineering, Faculty of Mechanical Engineering and Process Engineering, Université des Sciences et de la Technologie Houari Boumediene, BP 32, Algiers 16111, Algeria; obaaloudj@usthb.dz

**Keywords:** central nervous system, echovirus, gene amplification, picornaviridae, rhabdomyosarcoma, viral infections

## Abstract

Enteroviruses (EVs) represent a major cause of viral meningitis, being responsible for nearly 1 billion infections each year worldwide. Several techniques were developed to obtain better diagnostic results of EV infections. Herein, we evaluated the efficiency of EV detection through isolation on both Rhabdomyosarcoma (RD) and Vero cell line cultures, conventional reverse transcription-polymerase chain reaction (RT-PCR) and real-time RT-PCR. Thus, 50 cerebrospinal fluid (CSF) samples belonging to patients suspected to have viral meningitis in northern Algeria were collected, anonymously numbered from 1 to 50 and subjected to the above-mentioned techniques for EV detection. Using real-time RT-PCR, 34 CSF samples were revealed to be positive for viral origin of meningitis (68%). Thirteen of them were positive when the conventional RT-PCR was used (26%), and only three samples gave positive results when the cell culture technique was used (6%). Surprisingly, two cell culture-positive CSF samples, namely, 31 and 39, were negative using RT-PCR directly on the original samples. However, they turned to be positive when amplification was carried out on their corresponding cell culture supernatant. The cell-cultured viral isolates were then identified by sequencing their viral genome’s VP1 regions. All of them were revealed to belong to the echovirus 27 strain. This investigation demonstrates that RT-PCR techniques are often more sensitive, accurate and much faster, providing reliable results within a clinically acceptable timeframe. However, viral isolation on cell cultures remains crucial to obtain enough viral load for serological tests or even to avoid the rare, but existing, false negative PCR.

## 1. Introduction

Human enteroviruses (HEVs) are very common pathogens. They are responsible for very frequent infections, since they are estimated at nearly 1 billion each year worldwide [1,2,3]. The genus *Enterovirus* (EV), of the family Picornaviridae, consists of small, non-enveloped, single-stranded ribonucleic acid (RNA) viruses (27 to 30 nm particles) of positive polarity [4,5,6,7,8] HEVs, including four species (HEV A, B, C and D) and grouping more than 120 serotypes [5,9,10,11]. EVs’ viral particles are known to be resistant outside the host. They are primarily transmitted through the fecal–oral route, meaning that the virus can be present in fecal matter and contaminate various sources, such as food, water and surfaces. However, HEVs are not limited to this mode of transmission. They can also be spread through respiratory secretions when infected individuals cough or sneeze. This dual transmission mechanism broadens the range of opportunities for the virus to infect new hosts [12,13,14].

The large diversity of EV species and their genetic variability allow them to infect different organs, provoking acute infectious syndromes of a neurological, respiratory, cutaneous, muscular, cardiac, maternal–fetal or perinatal nature [5,10,15,16,17]. Moreover, HEVs represent one of the main causes of viral infections of children and adults through their gastrointestinal tracts, causing mild symptoms [18,19]. However, several EVs can invade the central nervous system (CNS) and cause severe pathologies such as meningoencephalitis, myelitis, paralysis, myocarditis, brain imaging abnormalities and long-term neurodevelopmental sequelae, where children with fragile immune defenses and an immature blood–brain barrier are at a higher risk of viral CNS infections and meningitis compared to adults [20,21,22,23,24,25]. EVs, notably non-polio human enteroviruses (NPHEV), are now recognized as the most common cause of acute meningitis, accounting for 80% to 92% of all aseptic cases in which the pathogen is identified. In addition, EV infection severity can vary from asymptomatic or mild cases to severe and life-threatening conditions [25]. This diversity in clinical outcomes underscores the importance of ongoing research and surveillance to better understand EV infections and develop effective prevention and treatment strategies for the various syndromes that they can provoke [26,27,28,29].

In order to determine the relationships between HEV infections and the various clinical syndromes, standard methods for EV detection and identification were developed over time. Initially, viral isolation on cell cultures, followed by serotyping through seroneutralization assays using specific antisera, were used [30,31,32]. Such procedures involve introducing a suspected virus-containing sample to specific cultured cells, allowing the virus to infect and replicate within the cells. This process aims to observe changes in the cell cultures that indicate viral presence, confirming the virus’s identity and enabling further study and characterization for diagnostic, therapeutic or research purposes [33]. Cell cultures are valuable in clinical and epidemiological investigations because they allow researchers and healthcare professionals to confirm the presence of HEVs in clinical samples and determine the specific serotype responsible for an infection, which is crucial for understanding the epidemiology of outbreaks and targeting vaccination efforts. Such techniques also correlate specific serotypes with particular clinical syndromes, helping to establish links between the virus and the associated diseases [32]. However, cell cultures are time-consuming and labor-intensive and require the availability of specific antisera for seroneutralization assays. Moreover, the genetic resemblance between HEVs and human parechoviruses (HPeVs), a group of viruses that used to belong to the EV family, may lead to confusing positive results when it comes to EV detection through cell culture techniques and hence are one of the main reasons for the misinterpretation of results and inaccurate conclusions about the presence of enteroviruses [34].

In addition, several techniques for rapid EV genome detection in clinical samples are based on the amplification of the 5′ non-coding genome region through the polymerase chain reaction technique (PCR). PCR is a cyclic process that creates an exponential increase in the DNA copies, enabling the detection, study and analysis of specific genetic material for various applications like diagnostics. Initially applied for amplifying DNA, its utility has expanded to include RNA studies, using a reverse transcriptase enzyme to generate complementary DNA (cDNA) from RNA, which is designated as reverse transcription PCR (RT-PCR) [35]. In EV diagnosis through PCR, viral RNA extraction from the sample, reverse transcription (RT), and cDNA amplification and detection are the common steps [30,36,37,38]. Yet, the PCR methods do not allow for viral serotyping and its genetic characterization. When serotyping is required, virus isolation on the cell culture remains the most appropriate technique to obtain a sufficient amount of the viral genome [39,40]. In order to overcome problems associated with the high antisera specificity, attempts were made to develop new methods for EV identification through PCR amplification and partial sequencing of specific regions of their genome, such as the VP1 region, coding for an enteroviral capsid protein [38,39,40,41].

Continuous monitoring, research and surveillance are crucial for understanding the epidemiology of HEVs and developing strategies to control their spread and manage outbreaks. In addition, the unmet gaps in the development of rapid and specific molecular analyses for a growing list of emerging and re-emerging neurotropic viruses is pushing toward the addition of new molecular assays and next-generation sequencing to enhance diagnostic abilities for identifying infectious meningitis. However, the expansion of test menus has led to new challenges in selecting appropriate tests and making correct interpretations [25]. Herein, we evaluated three techniques’ efficiency, namely, (1) cell culture, (2) conventional RT-PCR and (3) real-time RT-PCR, for EV detection in 50 cerebrospinal fluid (CSF) samples belonging to aseptic meningitis-diagnosed patients from northern Algeria. The objective was to determine if one of the three methods could constitute, on its own, an efficient alternative for EV detection in all of the studied samples. It is important to mention that real-time RT-PCR is a molecular technique that is used to amplify and simultaneously quantify a targeted DNA or RNA molecule. It is commonly used to detect, identify and measure the amount of a specific RNA molecule in a sample [42].

## 2. Materials and Methods

The work was carried out between February and June 2019 at the *Enterovirus* laboratory-WHO National Reference Laboratory for polio surveillance in Algeria. Thus, fifty CSF samples were taken from patients presenting viral meningitis symptoms. Sampling was carried out using lumbar punctures between the third and the fourth or between the fourth and the fifth lumbar vertebrae. Throughout the study period, fifty CSF samples were named using Arabic numerals from 1 to 50 and transported to the laboratory at 4 °C. EV presence in the samples was inspected using isolation on cell cultures, conventional RT-PCR and real-time RT-PCR.

### 2.1. Cell Culture

EV isolation through cell cultures was carried out by inoculation, in duplicate for each sample, on both Rhabdomyosarcoma (RD) and Vero cell lines derived from human Rhabdomyosarcoma and kidney epithelial cells of an African green monkey, respectively. Both RD and Vero cell lines are known to be sensitive to infection by enteroviruses.

As recommended by the World Health Organization Polio Laboratory Manual [43], 200 µL of each specimen was added to 2 mL RD/Vero cell-containing tubes. The tubes were previously prepared at a concentration of about 10^5^ cells/mL and incubated at 36 °C for 48 h before inoculation. After inoculation, the tubes were incubated at 36 °C for 7 days (passage 0: P0), allowing for the observation of a complete cytopathic effect (CPE) under an inverted microscope. Uninfected tubes from each of the two cell lines were used as negative controls. If no CPE appeared after 7 days, a blind passage (P0+1) was performed through inoculation of new cell culture-containing tubes with 200 µL of the old ones.

### 2.2. Conventional and Real-Time RT-PCR

#### 2.2.1. RNA Extraction

The QIAamp viral RNA mini kit (Qiagen, Tokyo, Japan) was used to isolate viral RNAs from 140 µL of each CSF specimen according to Casas et al. [44]. The elution step was performed with 60 µL of molecular-grade sterile water, and the extracted RNA was either used or stored at −80 °C for further analysis.

#### 2.2.2. Conventional RT-PCR

Complementary DNA synthesis was carried out in a 20 µL reaction mixture containing 3.5 µL of buffer (×10); 3.5 µL of MgCl_2_ (50 mM); 1.25 µL of rH (random hexamer); 1 µL of dNTP (10 mM); 1 µL of M-MLV reverse transcriptase (200 U/µL); 4.75 µL of sterile H_2_O (RNase free); and 5 µL of the extracted RNA.

Reverse transcription was realized in a thermocycler at 37 °C for 1 h. The RNA-DNA hybrids were then denatured, and the reverse transcriptase was inactivated by thermal shock (2 min in ice). The final cDNA product was be stored at −20 °C or directly amplified by PCR.

PCR was carried out using 1 μL of the obtained cDNA together with 24 μL of the reaction mixture: 12.5 μL of Taq polymerase; 0.5 µL of each EV-F and EV-R primers (Table 1) and 10.5 µL of H_2_O. The mixture was then subjected to the Applied Biosystems 2720 thermal cycler for 35 amplification cycles (30 s at 94 °C for denaturation, 30 s at 55 °C for hybridization and 45 s at 72 °C for elongation followed by a long elongation of 7 min). The final product was electrophoresed on a 2% agarose gel.

#### 2.2.3. Real-Time RT-PCR

Real-time RT-PCR was carried out using the ABI 7500 “Applied Biosystems” thermal cycler. The “One-step RT-PCR (Invitrogen, Waltham, MA, USA)” kit was used to carry out RT-PCR reactions using primers complementary to the 5′NC region (Table 2). The process allows for cDNA synthesis and amplification in one step. The reaction mixture was composed of 10 µL Master Mix One-step RT-PCR; 0.6 µL sense primer and 1.7 µL anti-sense primer (Table 2); 0.4 µL of probe and 2.3 µL H_2_O. The reaction mixture was dispensed at a rate of 15 μL per tube, to which 5 μL of the extracted sample was added until a total volume of 20 μL. Real-time RT-PCR was carried out as follows: reverse transcription for 30 min at 50 °C, then a denaturation step for 5 min at 95 °C. The amplification step was carried out in 40 denaturation cycles of 10 s at 95 °C and hybridization for 30 s at 60 °C.

#### 2.2.4. PCR Product Analysis

The PCR product analysis was realized through electrophoretic migration conducted on 2% agarose gel, supplemented with 2–3 µL SYBR Safe DNA Gel Stain for revelation and left for solidification at room temperature. The migration was carried out for 50–60 min at 80 V and the amplified fragments were visualized using an ultra-violet transilluminator and identified by comparing bands sizes to those of the molecular weight marker and the positive/negative controls.

### 2.3. Viral Serotype Identification

In order to determine their serotypes, EVs from the cell culture-positive samples were molecularly identified through their VP1 region sequences that were amplified (primers in Table 3) and sequenced. Thus, enzymatic sequencing was carried out according to the Sanger and Coulson method [47].

The obtained sequences were aligned using MEGA-6 (Molecular Evolutionary Genetics Analysis Version 6.0), then compared to the available sequences from the National Center for Biotechnology Information, NCBI: http://www.ncbi.nlm.nih.gov (accessed on 6 May 2019) using the BLAST algorithm (basic local alignment search tool).

### 2.4. Sensitivity Evaluation

According to [48] and references therein, test sensitivity, also identified as “true positive rate”, identifies how well a test can classify subjects who truly have the condition of interest (the viral meningitis). In our study, positive PCRs that were obtained from the cell cultures, but not directly from CSF samples, were considered as negative PCRs.

*Sensitivity* (*S*) = [*True positives* ÷ (*True positives* + *False negatives*)]

## 3. Results

### 3.1. Cell Culture

**Figure 1:**
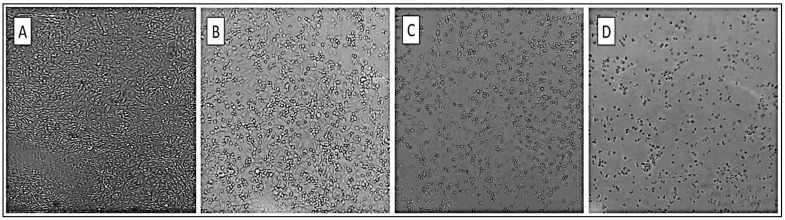
Cytopathic effect (CPE) of EV on RD cells. (**A**) No CEP; (**B**) 50% ECP on cells infected with the virus; (**C**) 75% ECP due to infection of all RD cells by the virus; (**D**) 100% ECP due to infection of all RD cells by the virus.

Among the 50 studied samples, only three positive results were obtained on the RD cell line (samples 9, 31 and 39). During the first seven days (P0), microscopic observation revealed no morphological differences between the inoculated cultures and negative controls. When blinded (P0+1), cells were rounded, became refractive and destruction of the cell mat was observed in infected cultures (Figure 1). However, none of the studied samples gave positive results on the Vero cell line.

### 3.2. Gene Amplification

#### 3.2.1. From Clinical Samples

**Figure 2:**
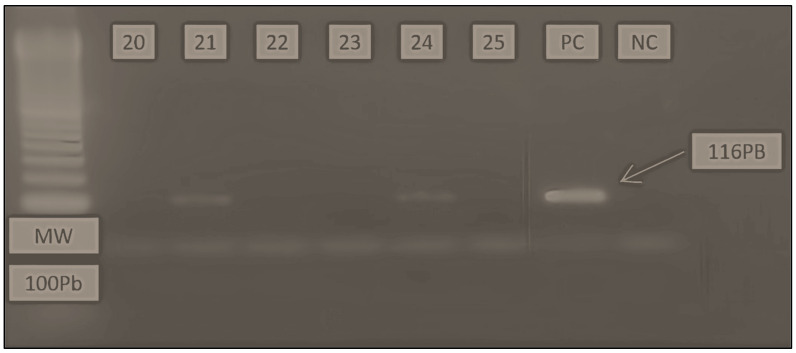
Electrophoretic migration on agarose gel (2%). The figure shows the amplification results of EV sequences from LCR samples using conventional RT-PCR. PC: positive control; NC: negative control. MW: molecular weight markers. Negative results (samples: 20, 22, 23 and 25). Positive results (samples: 21 and 24).

Conventional RT-PCR allowed us to detect the viral genome in 26% of the studied samples (13). Some of them showed low band intensity compared to the positive marker. In addition, the EV1/EV2 primers were the most efficient for viral genome amplification through this technique. It made it possible to obtain an amplified fragment of 116 bp with a hybridization temperature of 55 °C (Figure 2).

**Figure 3:**
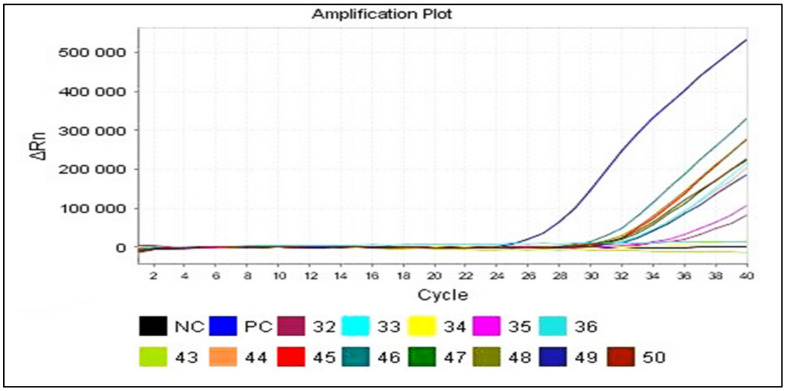
Amplification plot screen displaying post-run amplification of the samples 32, 33, 34, 35, 36, 43, 44, 45, 46, 47, 48, 49 and 50. Results obtained from real-time RT-PCR performed on the clinical samples. The fluorescence increases exponentially within each amplification cycle. In this example, the positive samples cross the detection threshold after 28–32 cycles. ΔRn: the magnitude of normalized fluorescence signal generated by the reporter at each cycle during the PCR amplification; PC: positive control; NC: negative control.

Real-time RT-PCR allowed for viral genome detection in 34 out of 50 CSF samples (68%), still using primers targeting the 5′NC region. In real-time PCR, the evolution of the fluorescence emitted is monitored during the amplification reaction using an indicator of amplicon production during each cycle **(**Figure 3). Therefore, in addition to the samples detected by conventional RT-PCR (13 samples), 21 other samples were also detected by real-time RT-PCR (Table 4). Table 5 represents the detailed results in terms of EV detection within the 50 CSF samples using cell culturing, RT-PCR and real-time RT-PCR.

#### 3.2.2. From Positive Culture Supernatants

**Figure 4:**
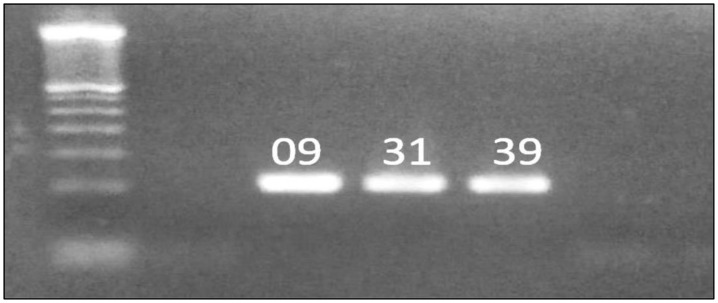
Conventional RT-PCR after gene amplification from the positive culture supernatant.

**Figure 5:**
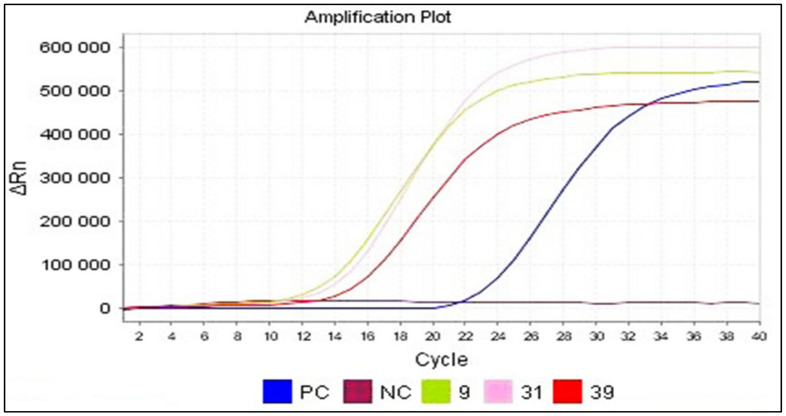
Amplification plot screen displaying post-run amplification of the samples 9, 31 and 39. Results obtained from real-time RT-PCR performed on the positive cell culture supernatant. The fluorescence increases exponentially within each amplification cycle. ΔRn: the magnitude of normalized fluorescence signal generated by the reporter at each cycle during the PCR amplification; PC: positive control; NC: negative control.

Among the three positive results obtained using the cell culture technique (9, 31 and 39), only two of them (31 and 39) have shown negative results using both RT-PCR when amplified directly from their CSF samples. A second amplification of those two samples, but this time using their positive cell culture supernatants instead of their CSF samples, allowed us to obtain positive results either through conventional RT-PCR (Figure 4), or real-time RT-PCR (Figure 5).

**Table 5:** Comparative results of EV detection by cell culturing, RT-PCR and real-time RT-PCR.

Samples	RD	Vero	PCR	Real T PCR	Samples	RD	Vero	PCR	Real T PCR
01	−	−	−	+	26	−	−	−	+
02	−	−	−	−	27	−	−	−	−
03	−	−	−	−	28	−	−	−	++
04	−	−	−	+	29	−	−	−	−
05	−	−	−	+	30	−	−	−	−
06	−	−	−	+	31	+	−	+*	+*
07	−	−	+	+	32	−	−	−	++
08	−	−	+	+	33	−	−	−	++
09	+	−	+	+	34	−	−	−	+/−
10	−	−	−	−	35	−	−	−	++
11	−	−	+	+	36	−	−	−	−
12	−	−	−	+	37	−	−	+/−	+
13	−	−	−	−	38	−	−	+	+
14	−	−	−	−	39	+	−	+*	+*
15	−	−	−	−	40	−	−	+/−	+
16	−	−	−	−	41	−	−	+/−	+
17	−	−	−	−	42	−	−	+/−	+
18	−	−	+/−	+	43	−	−	−	−
19	−	−	−	+	44	−	−	−	++
20	−	−	−	++	45	−	−	−	++
21	−	−	+	+	46	−	−	−	++
22	−	−	−	++	47	−	−	−	++
23	−	−	−	++	48	−	−	−	++
24	−	−	+	+	49	−	−	−	++
25	−	−	−	−	50	−	−	+/-	++

RD: cell culturing on RD line; Vero: cell culturing on Vero line; PCR: conventional RT-PCR; Real T PCR: real-time RT-PCR; (−) negative; (+/−) weakly positive; (+) positive; (++) strongly positive. (+*) positive only when PCR was realized on the cell culture supernatant.

### 3.3. Molecular Identification of the Cell Culture-Isolated Strains

The cell culture-isolated strains were molecularly identified through sequencing of their VP1 region. Sequence analysis revealed that our isolates belong to echovirus 27 (HEV-B).

### 3.4. Sensitivity Evaluation

According to Table 5, cell culturing on the RD cell line showed 3 positives, while conventional and real-time RT-PCR gave 13 and 34 positives, respectively. The total number of positive results obtained using the three techniques were 36. Thus, the three techniques’ sensitivities are measured as follows:

S1 = [3 ÷ (3 + 33)] = 0.083 (8.3%)

S2 = [13 ÷ (13 + 23)] = 0.361 (36.1%)

S3 = [34 ÷ (34 + 2)] = 0.944 (94.4%)

S1, S2 and S3 represent the cell culture, conventional RT-PCR and real-time RT-PCR sensitivity, respectively. The results herein show that conventional and real-time RT PCR are more sensitive to EV presence in CSF samples and thus allowed us to detect ca. 36 and ca. 94% of the total abstained positives, respectively.

## 4. Discussion

In this study, both RD and Vero cell lines have been used for EV cultivation from CSF samples. The objective of using two different cell lines is to increase the chance of isolating the virus, since there is no cell line that is known to be receptive to all EVs [49]. The RD cell line seems to be more sensitive to EVs from our samples compared to Vero cells. Similar results were obtained by Melnick [50]. Moreover, several studies showed that EVs develop well on RD cells, Hep-2 cells (human epithelial cells type 2) and MRC-5 cells (human embryonic cells). Similarly, Refs. [1,51] showed that RD cell lines were the most sensitive to EV isolation.

In our study, only 3 CSF samples were positive for EV detection on the RD cell line and none of the 50 CFS samples gave positive results on the Vero cell line. Similar results were observed in the study in [52], where only 10 out of 116 samples (8.6%) gave a positive result. This low detection sensitivity is due to several reasons such as (1) the sampling in relation to the disease stage and therefore the sample’s load in terms of active viral particles; (2) the loss of viral viability due to improper delivery of CSF samples, considered as a fragile liquid. According to [53], CSF samples’ transport at ambient temperatures (>4 °C) leads to a loss of EV viability. Thus, prolonged transport will mainly affect the cell culture results of viruses in samples with low viral load, especially easily alterable samples such as CSF samples. Moreover, (3) the growth and propagation difficulty of certain EVs in cell cultures may also be the cause. According to Harvala et al. [29], EVs belonging to the species A, including EV-A71, are characterized by poor growth on cell cultures. Meanwhile, EV-D68 requires incubation temperatures lower than those normally applied for EV cultivation. In addition, Ref. [54] demonstrated that coxsackievirus types A1–A6 cannot be cell-cultured.

The cell lines’ sensitivity toward EVs (presence of viral receptors) is also one of the major reasons for the false negative results in EV cultures. EVs are likely to recognize a wide range of membrane receptors. Some of them are specific to a small number of EVs, while others seem to be ubiquitous and are used by several genotypes [55]. In this context, Ref. [51] used both RD and Hep-2 cell lines for EV cultivation from CSF samples. Their results demonstrated that RD cells are more sensitive to echoviruses and coxsackie virus A. However, Hep-2 cells are remarkably more sensitive to coxsackie virus B. Furthermore, Ref. [56] demonstrated that Vero cells are more sensitive to coxsackie virus B compared to CMK cells (primary cynomolgus monkey kidney cells) and that echovirus- and coxsackie virus-containing samples were all positive on CMK cells, but only 35% of them were positive on Vero cells.

In our study, it seems evident that real-time RT-PCR was, so far, more efficient for EV detection in CSF samples compared to conventional RT-PCR, and both of them were more effective than cell cultures. In fact, real-time RT-PCR uses different primers that target different sequences from the 5′NC region. According to [45,57,58], the differences between the primer and/or probe sequences in conventional RT-PCR and the EV type are responsible, in some cases, for false negative results. Impressively, the cultural methods in our study could cultivate some similar viruses such as HPeVs, while the PCRs appears to be negative for the same samples [34].

In addition, the detection system in real-time RT-PCR is equipped with specific probes that hybridize at the same time as the primers, allowing for a more sensitive detection, even of a low level of amplicons [59]. Otherwise, conventional RT-PCR allows for visual detection only after electrophoretic migration, where a low amplicon load could be invisible and a positive amplification could not be detected. Also, the absence of a post-PCR step in the real-time RT-PCR accelerates the results, limiting contaminations, decantation and a loss of viral particles [59]. Real-time RT-PCR has already been shown to be highly sensitive for EV detection. Archimbaud et al. [60], for example, compared the efficiency of conventional and real-time RT-PCR techniques for SV detection in CSF samples of patients with clinical signs of viral meningitis. Their results demonstrated that real-time RT-PCR was as sensitive as conventional RT-PCR.

On the other side, EV genome detection in CSF samples through PCR techniques proved to be more sensitive than traditional cell cultures for a rapid diagnosis of EV meningitis. In this context, Ref. [53] showed that 80% of negative cell cultures were positive through RT-PCR, which confirms the great sensitivity of these techniques toward viruses with difficult propagation on cell lines and/or non-cultivable viruses. Dahee et al. [26] also confirmed the RT-PCR has high sensitivity compared to cell cultures for EV meningitis diagnosis. They demonstrated that only 32.3% of RT-PCR positive samples were positive on cell cultures, while all of the other samples (67.7%) showed no significant CPE on cell lines.

The other inconvenience of cell culturing techniques is the fact that they can only detect viable viral particles, while molecular techniques are able to give positive results from samples with a very low load of a viral genome. According to Read et al. [54], viral detection through RT-PCR is probably more reliable than viral culture, since it does not require a viral replication competence and can also detect uncultivable viruses. In addition, RT-PCR is a quick and precise method for diagnosing EVs, detecting them within 24 h [39]. Hence, PCR proved its efficiency as a rapid and sensitive alternative to cell cultures for the diagnosis of central nervous system infections caused by EVs [45,46,53].

Impressively, it is important to mention that the three positive samples through cell cultures did not all give positive results using conventional RT-PCR. The negative RT-PCR results in the two samples, 31 and 39, could be due to the low viral load in the CSF [53]. The false negative results may also be due to inhibitory substances that interfere with the function of PCR enzymes [53]. It is also likely that technical issues such as viral RNA degradation could be implicated in the PCR low sensitivity to some clinical samples [61].

According to [62], an initial viral culture could allow for the multiplication of the rare EVs initially present in CSF, thus enhancing their viral load and increasing the probability of encounter (primer–genome) in PCR techniques. Such interpretation could explain the positive amplification results obtained from a culture supernatant instead of the direct sample using.

From this study, it appears evident that in some viral meningitis cases, combining both cell cultures and amplification techniques is the only way to avoid false negative results. However, we cannot deny that real-time RT-PCR is superior to viral culture of CSF for the diagnosis of EV meningitis and that the utility of culturing viruses from CSF is limited. Real-time RT-PCR is more sensitive and reliable as it allows for a positive diagnosis with minimal delay and may thus influence clinical decisions.

EVs’ genome region that encodes the major capsid protein VP1 is the most variable among EV populations and has the main antigenic neutralization sites [63,64]. VP1 sequence analysis revealed that the three isolates, obtained through cell cultures, were identified as echovirus 27 (HEV-B). This strain belongs to the NPHEV, known as the major cause of all cases of aseptic meningitis in which the pathogen is identified [27,28,65]. According to Ibrahim [64], the high variability in the selected region (VP1) for sequencing, the use of degenerated primers in the first amplification and the technique sensitivity are not sufficient to allow for the direct strains’ characterization from clinical samples, particularly CSF samples.

The results herein underscore the challenges associated with diagnosing EV meningitis. The use of different cell lines for viral cultivation from cerebrospinal fluid samples highlights the need for the optimization of cell-based methods in virology by integrating molecular techniques to obtain increased sensitivity, but also for a diverse approach due to the absence of a “universally receptive cell line” for all EVs [33]. On the other hand, real-time reverse transcription-polymerase chain reaction (RT-PCR) emerges as a more efficient and sensitive tool for EV detection compared to conventional RT-PCR and viral cell cultures [66,67]. However, limitations such as the timing of sample collection, delivery conditions, viral load and the difficulty in propagating certain EVs in cell cultures can contribute to false negative results [33]. Importantly, the ability of RT-PCR to detect viral genomes, even in samples with very low viral loads, and its speed of diagnosis make it a valuable alternative to traditional cell cultures for rapid EV meningitis diagnosis. Nonetheless, in select cases, a combined approach involving both cell cultures and amplification techniques may be necessary to mitigate false negatives. It is crucial to recognize the limitations of each diagnostic method and tailor the approach to maximize the accuracy of EV meningitis diagnosis, ultimately influencing clinical decisions for patient management. Furthermore, the genetic variability in VP1 in EVs emphasizes the challenges in directly characterizing strains from clinical samples, highlighting the importance of a comprehensive diagnostic approach in managing these infections [24,68].

In the last few years, new emerging approaches for EV detection in CSF are being developed, such as the identification of biological markers in CSF that are associated with EV infection and next-generation sequencing analysis of amplicons covering the entire capsid coding region directly synthesized from clinical samples. However, those techniques require more time to be validated and are still waiting for more scientific and technical approvals and hence are not adopted yet in clinical analysis [24].

In the last few years, the COVID-19 pandemic has drawn global attention to the dangers posed by viruses to humanity. This health crisis has highlighted the significance of the need for a swift and coordinated global response and the substantial impact that viral infections can have on public health, economies and individuals’ daily lives [69]. In the same context, and despite the fact that the COVD-19 pandemic appeared after the achievement of this work, it seems important to mention that cases of viral meningitis due to COVID-19 have been recorded, where fever was the most common symptom, followed by headaches, cough and vomiting/nausea [70]. Moreover, it appears that cases of aseptic meningitis may occur after mRNA-based vaccination against COVID-19 [71]. In general, the COVID-19 pandemic, but also the increasing concerns of infections by enteroviruses (EVs) causing severe disease in humans, constituted a real alarm to both researchers and pharmaceutical manufacturers, signaling the urgency to develop more rapid and effective diagnosis, treatment and vaccination strategies against viral infections [68].

Overall, limitations such as the variation in cell line sensitivity, with RD being more sensitive than Vero, highlights the challenge of achieving universal receptivity for all enteroviruses. Additionally, the low detection sensitivity in cell cultures, potential false negatives in PCR techniques and the limitations of VP1 sequencing for strain characterization underscore the complexities in diagnosing EV infections. The impact of factors such as sampling time, delivery conditions and the difficulty in propagating certain enteroviruses in cell cultures further contribute to the limitations. While real-time RT-PCR is recognized as superior, the need for a combined approach in certain cases and the emergence of new diagnostic techniques, still in the validation stage, add layers of complexity to this study’s scope.

## 5. Conclusions

This study reveals the intricate challenges associated with diagnosing EV meningitis. The absence of a universally receptive cell line for all EVs necessitates a diverse approach, as highlighted by the use of different cell lines for viral cultivation from cerebrospinal fluid samples. Real-time RT-PCR emerges as a more efficient and sensitive tool for EV detection compared to conventional RT-PCR and viral cell cultures. However, limitations such as sample collection timing, delivery conditions and the difficulty in propagating certain EVs in cell cultures can contribute to false negative results. While RT-PCR’s ability to detect viral genomes, even in samples with low viral loads, and its rapid diagnosis capability make it a valuable alternative to traditional cell cultures, in select cases, a combined approach involving both cell cultures and amplification techniques might be necessary to mitigate false negatives. Recognizing the limitations of each diagnostic method and tailoring the approach is crucial to maximize the accuracy of EV meningitis diagnosis, influencing clinical decisions for patients’ disease management.

## Figures and Tables

**Table 1:** Primers used for conventional RT-PCR.

Primers	Genome Region	Sequence	Location	Reference
EV2	5′NC	5′-TCCGGCCCCTGAATGCGGCTAATCC-3′	446–470	
EV1	5′NC	5′-ACACGGACACCCAAAGTAGTCGGTCC-3′	559–533	[45]

**Table 2:** Primers used for real-time RT-PCR.

Primers	Genome Region	Sequence	Location	Reference
Vrp F	5′NC	5′-CCCTGAATGCGGCTAATCC-3′		
Vrp R	5′NC	5′-ATTGTCACCATAAGCAGCCA-3′	452–596	[46]
Probe	5′NC	5′-AACCGACTACTTTGGGTGTCCGTGTTTC-3′		

F: forward; R: reverse.

**Table 3:** VP1 region primers used for molecular identification.

Primer	Genome Region	Sequence	Location	Reference
AN89	VP1	CCAGCACTGACAGCAGYNGARAYNGG	2602-2627	
AN88	VP1	TACTGGACCACCTGGNGGNAYRWACAT	2977-2951	
AN232	VP1	CCAGCACTGACAGCA	2602-2616	
AN233	VP1	TACTGGACCACCTGG	2977-2963	[40]

Degenerate primers: Y = C, T; R = A, G; W = A, T; N = A, C, G, T.

**Table 4:** Comparison of the results obtained by conventional and real-time RT-PCR.

		Conventional RT-PCR			
		Positive		Negative	Total
Real-time RT-PCR	Positive	13	+	21	50
		+		+	
	Negative	0	+	16	
	Total	50			

## Data Availability

Data are contained within the article.
